# Genotypic-specific hormonal reprogramming and crosstalk are crucial for root growth and salt tolerance in bermudagrass (*Cynodon dactylon*)

**DOI:** 10.3389/fpls.2022.956410

**Published:** 2022-08-04

**Authors:** Yong Yang, Misganaw Wassie, Ning-fang Liu, Hui Deng, Yi-bing Zeng, Qian Xu, Long-xing Hu

**Affiliations:** ^1^College of Physical Education, Changsha University, Changsha, China; ^2^Department of Pratacultural Sciences, College of Agronomy, Hunan Agricultural University, Changsha, China; ^3^CAS Key Laboratory of Plant Germplasm Enhancement and Specialty Agriculture, Wuhan Botanical Garden, Chinese Academy of Sciences, Wuhan, China; ^4^Grassland Research Center of Hunan Province, Changsha, China

**Keywords:** bermudagrass, gene expression, hormone, root growth, salt stress

## Abstract

Salt stress is one of the major abiotic factors limiting the productivity of bermudagrass (*Cynodon dactylon*). However, the role of hormonal reprogramming and crosstalk in regulating root growth and salt tolerance in bermudagrass was not reported. Here, we examined the physiological and hormonal responses of two contrasting bermudagrass genotypes; ‘C43,’ salt-tolerant ‘C198’ salt-sensitive. Under salt stress, ‘C43’ had better membrane stability and higher photosynthetic activity than the ‘C198.’ Salt stress promoted root growth and improved root/shoot ratio and root activity in ‘C43,’ but the root growth of ‘C198’ was inhibited by salt stress, leading to diminished root activity. The two bermudagrass genotypes also showed critical differences in hormonal responses, especially in the roots. The root contents of indole-3-acetic acid (IAA), cytokinin derivatives, such as *trans-*zeatin riboside (*t*ZR) and dihydrozeatin riboside (DHZR) were increased in ‘C43,’ but decreased in ‘C198’ when exposed to salt stress. The root growth rate was positively correlated with the root IAA, tZR and DHZR, indicating their crucial role in root growth under salt stress. The expressions of *TAA*/*YUCCA* and *CYP735A* involved in IAA and tZR biosynthesis were induced by salt stress in ‘C43,’ but inhibited in ‘C198,’ leading to reduced hormone accumulations. Salt stress decreased the iP, tZ, and DHZ content in the roots of both genotypes, and no significant difference was observed between the two genotypes. Salt stress reduced the content of GA_3_ in both genotypes by inhibiting *GA20ox* and *GA2ox* genes, which could be attributed to the reduced shoot growth in both genotypes. The increased ABA level by salt stress was significantly higher in ‘C198’ than ‘C43.’ Furthermore, there were positive and negative correlations between different hormones and root growth, suggesting that root growth could be regulated by complex hormonal reprogramming and crosstalk. This study provides a foundation for understanding the underlying mechanisms of hormonal-mediated root growth and salt tolerance in bermudagrass.

## Introduction

Salt stress is a severe global problem that adversely affects plant growth, development, and productivity (Munns and Tester, 2008; Gupta and Huang, 2014). The primary effects of salt stress are caused by osmotic stress, which results from the accumulation of ions in the rhizosphere (Ismail et al., 2014). The accumulation of ions (mainly Na^+^) in the plant cells further causes ion toxicity and inhibits various developmental processes (Munns and Tester, 2008; Deinlein et al., 2014). Salt stress induces the overproduction of reactive oxygen species (ROSs), including H_2_O_2_ (hydrogen oxide), ^⋅^O_2_^–^ (superoxide anion radical), ^⋅^OH (hydroxyl radical) in different organelles and results in oxidative stress (Munns and Tester, 2008), which further disturbs cellular metabolism, and inhibit enzyme activity and photosynthesis, and cause an eventual death of plants (Yu D. et al., 2020).

Roots are the essential organs that absorb water and nutrients from the soil and supply them to the shoots (Zou et al., 2021). Depending on the severity of the stress and plant species, salt stress could impair root development by inhibiting root length and root branching (Gupta and Huang, 2014; Zou et al., 2021). However, root growth can be promoted under salt stress in halophytes (Rahman et al., 2021) and a few salt-tolerant plant species, such as bermudagrass (Hu et al., 2012, 2015). Such responses could be one of the strategies for salt stress acclimation in plants. When exposed to salt stress, plants undergo a dynamic change in the root system architecture to maintain growth and development, thereby enhancing salt tolerance (Zou et al., 2021). Root system architecture modification, such as decreasing lateral root initiation, limiting root growth rates, and redirection of root growth, are crucial mechanisms to cope with osmotic stress and maintain normal root activities under salt stress (Zou et al., 2021). Salt stress-induced increase in root/shoot ratio as a result of improved root growth and inhibition of shoot growth (Albacete et al., 2008) could be an adaptive response to recover the functional equilibrium between below- and above-ground organs, which allows the roots to obtain more water and nutrients from the root zone in saline soils (Pérez-Alfocea et al., 2010).

As a sessile organism, plants develop various complex tolerance mechanisms to cope with the adverse effects of salt stress at the morphological, physiological, biochemical, and molecular levels (Gupta and Huang, 2014). Different metabolites and hormones are produced in different organs to provide stress tolerance mechanisms. Roots are sites for the biosynthesis of various hormones and metabolites involved in salt tolerance (Lu et al., 2019).

Phytohormones regulate plant growth and development and abiotic stress responses (Ryu and Cho, 2015; Yu Z. et al., 2020). The five major plant hormones are ethylene, ABA, gibberellin (GA), auxins such as indole-3-acetic acid (IAA), and cytokinin (CK). These hormones could play vital roles in the salt tolerance of plants individually or collectively through hormonal crosstalk (Ryu and Cho, 2015; Yu Z. et al., 2020). Auxin is a crucial hormone involved in almost all stages of plant development in all organs (Wolters and Jürgens, 2009). IAA is the most active form of auxin that play a crucial role in plant growth and development, including stress response (Cao et al., 2019). ABA is recognized as a stress hormone involved in stress adaptation by inducing stomatal closure and reducing water loss (Mehrotra et al., 2014; Sah et al., 2016). Additionally, ABA could enhance root growth in a dose-dependent manner; a higher concentration inhibits root elongation while promoting root elongation at a moderate concentration (Ghassemian et al., 2000). ABA inhibited the root growth of Arabidopsis by increasing ethylene biosynthesis (Luo et al., 2014).

Cytokinin is another essential plant hormone involved in plant growth and development. CK derivatives, such as tZR and iPA facilitate the responses to delay both stomatal closure and leaf senescence under abiotic stresses, and early CK-induced metabolic disorder may be one of the reasons for the inhibition of shoot growth and leaf senescence under abiotic stress (Ryu and Cho, 2015). CKs can also play positive and negative roles in the stress tolerance of plants (Hai et al., 2020). GA is a critical growth hormone involved in seed germination, stem and root elongation, leaf expansion, flower, and seed development (Ryu and Cho, 2015). GA stimulates root growth and development by monitoring cell proliferation and elongation. The deficiency in endogenous GAs could cause the development of shorter roots and smaller root meristems (Achard et al., 2009).

Hormonal crosstalk is crucial to regulating plant growth and development (Pacifici et al., 2015). For example, ABA plays a role in root growth with auxin crosstalk (Wang et al., 2017; Sun et al., 2018). Auxin could interact with cytokinin and negatively regulate cytokinin accumulation by rapidly inhibiting cytokinin biosynthesis. In contrast, cytokinin overproduction had a slower effect on auxin to regulate plant development (Nordstrom et al., 2004). Despite the role of these hormones have been studied in different plant species (Ryu and Cho, 2015), how these hormones are reprogrammed and the role of their complex interactions in regulating root growth and salt tolerance in bermudagrass was not reported.

Bermudagrass (*Cynodon dactylon* (L). Pers.) is a warm-season turfgrass species widely used to establish sports fields, lawns, and golf courses (Pessarakli, 2015). The growth and development of bermudagrass are affected by different abiotic stresses, including salt stress (Hu et al., 2015). Our previous study showed that improving the root/shoot ratio in plants exposed to salt stress is one of the salt tolerance mechanisms in bermudagrass (Hu et al., 2012). However, the detailed mechanisms associated with the differences in root growth and salt tolerance in bermudagrass, especially at hormonal level, were not reported. Because roots are the sites of hormone synthesis, we hypothesized that the difference in root growth between salt-tolerant and salt-sensitive bermudagrass genotypes could be related to the accumulation of different phytohormones and their specific crosstalk.

In this study, we used liquid chromatography coupled with MS (LC-MS) to characterize the accumulations of different phytohormones in two contrasting bermudagrass genotypes (‘C43,’ salt-tolerant; ‘C198’ salt-sensitive) in response to salt stress. Furthermore, to understand how phytohormones are regulated at the molecular level, we investigated the mRNA expression levels of critical genes involved in different phytohormones’ metabolism. Overall, this study shed light on how hormonal reprogramming and crosstalk are involved in root growth and salt tolerance in bermudagrass.

## Materials and methods

### Plant materials and growth conditions

Plant materials were prepared according to our previous studies (Hu et al., 2012, 2015). Briefly, uniform stolons with two nodes (5 cm long) of bermudagrass, ‘C43’ (salt-tolerant) and ‘C198’ (salt-sensitive), were planted in solid growth substances (2 peat soil: 1 sand, v/v). Two weeks after planting, an equal amount of plants was transplanted to plastic pots (7 cm diameter and 9 cm tall) filled with coarse silica sand as the plant anchor medium. Pots were suspended over tubs containing 46 L of aerated half-strength Hoagland solution (Hoagland and Arnon, 1950). The tubs were refilled every other day and renewed weekly. The bottom of the pots had a coarse nylon screen to allow the root growth into the solutions. Plants were grown in an environmentally controlled walk-in growth chamber with a temperature regime of 30/25°C (day/night) and photosynthetically active radiation levels of 800 μmol m^–2^ s^–1^ at canopy height for 14 h for 4 weeks. During the establishment period of shoot and root, the shoots were cut weekly at 4-cm height, and roots were clipped back to the bottoms of the pots at the beginning of the salt treatment to allow the plants to reach full maturity and develop uniform and equal size roots and shoots.

### Treatment and experiment design

After 4 weeks of cultivation, plants were subjected to 0 mM (control), 180 mM and 300 mM NaCl (salt stress) for 10 days and then canopy height and root length were determined. Roots elongated from nylon screen into nutrient solution were sampled after 4 h light. Plants were removed from the nutrient solution and roots were gently washed for 30 s with distilled water. The root tips, encompassing the meristem and the elongation zone, were excised with a scalpel from the remaining root system and immediately frozen in liquid nitrogen and stored at −80°C for further analysis. Four independent biological replicates, each containing a pool of twenty different plants, were sampled for hormone and qPCR analysis. The salt treatments and grass genotypes were arranged in a randomized complete block design with four replicates.

### Measurements

#### Shoot and root growth rate analysis

Shoot and root growth rates were determined as the difference in growth before and after salt treatment (Hu et al., 2012). The root growth rate was determined only for roots extending from the nylon screen at the pot bottom.

#### Root viability

The root activity was detected by the TTC method as described previously (Lutts et al., 2004). Briefly, 0.5 g of fresh roots were cut into pieces and quickly rinsed in deionized water containing 0.06% Tween 20 and incubated at 30°C in darkness in tubes containing 6 ml of 0.5% TTC 50 mM K_2_HPO_4_, pH 7.0 for 20 h. The colored root samples were blotted dry and rinsed in deionized water, then incubated in 10 ml of 95% ethanol at 60°C for 4 h under gentle agitation during the extraction. The absorbance of the extract solution was recorded at 530 nm and root activity was calculated according to the standard curve with the following equation:

Rootactivity=D/(W×t)[μg/(g.h)].

Where *D* represents the deoxidizing amount of the TTC (mg); *W* is the fresh weight of roots (g); *t* stands for the time of coloration (h).

#### Cell membrane stability

Cell membrane stability was determined as electrolyte leakage (EL) according to our previous study (Hu et al., 2009). Briefly, about 0.1 g plant tissues were washed with deionized water three times and placed into plastic tubes filled with 15 ml deionized water after being cut to 1 cm long segments and then shaken for 24 h. The initial conductivity (*C*_i_) was determined using a conductivity meter (JENCO-3173, Jenco Instruments, Inc., San Diego, CA, United States). Subsequently, the tubes were autoclaved at 121°C for 20 min, and the second conductivity (*C*_max_) was determined after the solution cooled to room temperature. Relative EL was calculated using the formula: EL (%) = (*C*_i_/*C*_max_) × 100.

#### Photosynthetic parameters

Photosynthetic gas exchange measurements were performed in the third fully expanded leaves using a portable infrared gas analyzer (Li-6400, LICOR, Inc., Lincoln, NE, United States) with the controlled atmosphere (400 μmol mol^–1^ CO_2_, 500 μmol s^–1^ flow rate) and a Licor 6400 LED external light source providing a photosynthetic photon flux density of 600 μmol m^–2^s^–1^. Net photosynthetic rate (*P*_n_) and stomatal conductance (*G*_s_) were measured for three subsamples in each pot, with each subsample consisting of five leaves (Hu et al., 2009).

#### Hormone extraction and fractionation for high performance liquid chromatography-mass spectrometry analysis

Hormones were analyzed as previously described (Albacete et al., 2008). Cytokinins (tZ, ZR, DHZ, DHZR, iP, iPA), auxins (IAA, IBA), gibberellic acid (GA_3_), and ABA were extracted and purified according to the method of Dobrev and Kamínek (2002). Briefly, about 0.5 g frozen tissues were ground into a fine powder using a pre-chilled mortar and pestle and then soaked in 5 ml of cold extraction mixture of methanol/water/formic acid (15/4/1, v/v, pH 2.5). After 24 h of extraction at −20°C, solids were separated by centrifugation at 12,000 ×*g* for 15 min, and re-extracted for 1 h in an additional 2 ml of the same extraction solution at 4°C. After centrifugation, extracts were combined with the first supernatant. Pooled supernatants were passed through a Sep-Pak tC18 cartridge (SepPak Plus, Waters, Milford, MA, United States) on an automated solid-phase extraction system (SPE215; Gilson, Middleton, WI, United States) to remove interfering lipids and plant pigments. The column was washed with 0.3 ml of extraction solvent and then combined with elutes. The combined elute evaporated in a vacuum concentrator at 40°C (MiniVac Beta, LABOGENE, Copenhagen, Denmark) and then reconstituted with 2 ml of 1 M formic acid. The hormone-containing fraction was passed through an Oasis MCX 96-Well Plate 150 mg (Waters) preconditioned with 2 ml of methanol followed by 2 ml of 1 M formic acid. After washing with 1 M formic acid, ABA and auxins were eluted with 2 ml of methanol, and cytokinins were eluted with 2 ml of 0.35 M ammonia in 60% (v/v) methanol as indicated in Dobrev and Kamínek (2002). Each fraction was evaporated to dryness in a vacuum concentrator, and the residues were dissolved in a 500 μl of water/methanol (70/30, v/v) mixture. Before injection, the dissolved fraction was filtered through 13-mm-diameter Millex filters with a 0.22-μm-pore nylon membrane (Millipore, Bedford, MA, United States) and the samples were transferred to 2-ml HPLC vials.

Analyses were carried out on a high-performance liquid chromatography (HPLC)-mass spectrometry (MS) system (Accela; Thermo Fisher Scientific, San Jose, CA, United States) coupled to a triple quadrupole mass spectrometer (TSQ Quantum Access MAX, Thermo Fisher Scientific, San Jose, CA, United States) and equipped with a heated electrospray ionization source (HESI-I). For each sample, 10 μl was injected into a Zorbax SB-C18 HPLC column (3.5 μm, 150 × 2.1 mm, Agilent Technologies) maintained at 35°C and eluted at a flow rate of 200 μl min^–1^. Mobile phase A consisted of water with 0.1%formic acid, and mobile phase B (methanol), was used for the chromatographic separation. The elution program maintained 70% A, 30% B for 3 min, then a linear gradient from 30 to 70% B in 7 min, followed by another linear gradient from 70 to 95% B in 10 min, and finally 95% B maintained for another 5 min. The column was equilibrated with the starting composition of the mobile phase for 30 min before each analytical run. The eluting ions were subjected to multiple reaction monitoring (MRM). The mass spectrometer was operated in the positive mode for cytokinins and IAA, and negative mode for ABA with a capillary spray voltage of 3500 V and a scan speed of 22,000 (m/z)/s from 50 to 600 m/z. Instrument control, data acquisition and processing were performed with Xcalibur 2.1 software (Thermo Fisher Scientific, San Jose, CA, United States). The level of plant hormones (tZ, t-ZR, DHZ, DHZR, iP, iPA, ABA, GA_3_, IBA, and IAA) in the samples were quantified using the calibration curves constructed for each analyzed component (0.5, 1.0, 2.0, 5.0, and 10.0 μg.ml^–1^) and corrected with the labeled forms of each compounded as internal standards at 0.1 μg.ml^–1^. The recovery percentages ranged between 90 and 92%.

#### qRT-PCR analysis of genes involved in hormone biosynthesis

Total RNA was extracted from fresh leaves and roots using the Trizol reagent (Invitrogen, Carlsbad, CA, United States) according to the user’s manual. After extraction, the RNA pellet was dissolved in 100 μl of RNase-free water. RNase-free DNase I was added to the total RNA to remove DNA contamination. The total RNA concentration was then determined by absorbance at 260 nm and RNA quality was evaluated on a 0.8% agarose gel. The first-strand cDNA fragments were synthesized from 2 μg of total RNA using oligo(dT)12-18 primer using a cDNA synthesis kit (Fermentas, Burlington, ON, Canada). The gene-specific primers (Table 1) were designed based on the target gene sequences using Primer 5 software (Hu et al., 2015). The actin gene was used as an internal standard. The qRT-PCRs were performed with ABI7500 in a final volume of 20 μl, with each containing 2 μl of cDNA, 10 μl of 2 × SYBR Green qPCR Mix (Takara, Otsu, Shiga, Japan) and 2 μM of the forward and reverse primers. Three independent biological replicates of each sample and two technical replicates of each biological replicate were used for real-time PCR analysis. The thermal cycling conditions were as follows: 40 cycles of 95°C denaturation for 5 s, and 52∼55°C annealing and extension for 20 s. After the PCR, a melting curve was generated by gradually increasing the temperature to 95°C to test the amplicon specificity. The relative expression of genes was calculated using the 2^–ΔΔCt^ method (Livak and Schmittgen, 2001).

**TABLE 1:** Primers used for real-time quantitative PCR in this study.

Gene name	Accession		Primers sequences (5′–3′)	Size (bp)	Tm (°C)
*NCED*	XP_008646127.2	F	CATTCCTCTTCCTCTTGTGT	122	55
		R	GTGTATGCTATGTTGCCTAG		
*ZEP*	XM_012847662.1	F	ATCTGTCTGTCCGAATAGTG	170	56
		R	CAGTTGGCGATGATGCTA		
*IPT*	XP_022682330.1	F	GTGGACGAGAGAGTTGGA	226	56
		R	GAACGCCGACAAGATACA		
*CKX*	XP_004970152.1	F	GTCTCGTCGTCGTAGTTC	232	58
		R	CGCTCTACTCCAACTTCTC		
*CYP735A*	OQU80479.1	F	CTCGTGGATCACCATCTG	244	58
		R	CAAGAAGAAGAACAGCAACA		
*TAA*	XP_002455113	F	GGCGACACCTACATTGAG	173	55
		R	GACACCGTGAAGAGCATAA		
*YUCCA*	PAN21149.1	F	CGAAGATGACAGCATTGAAG	128	52
		R	CGACATTGGAGCACTAACA		
*GA2ox*	XP_004953177.1	F	CGAAGTAGATGAACGATAGC	199	53
		R	CTCAGGTCCAACTGCATT		
*GA20ox*	XP_002463483.1	F	ACCACCTTGTCCATCTCC	143	53
		R	GCCTTCGTCGTCAACATC		
*Actin*		F	TCTGAAGGGTAAGTAGAGTAG		
		R	ACTCAGCACATTCCAGCAGAT		

### Statistical analysis

All data were subjected to two-way ANOVA(analysis of variance) according to the general linear model procedure of SAS (SAS Institute, Cary, NC, United States) to determine the effects of genetypes, salinity and their interactions. Treatment means were separated using the Duncan’s multiple range test at the *P* = 0.05 level of probability.

## Results

### Phenotypic responses of bermudagrass to salinity stress

**FIGURE 1:**
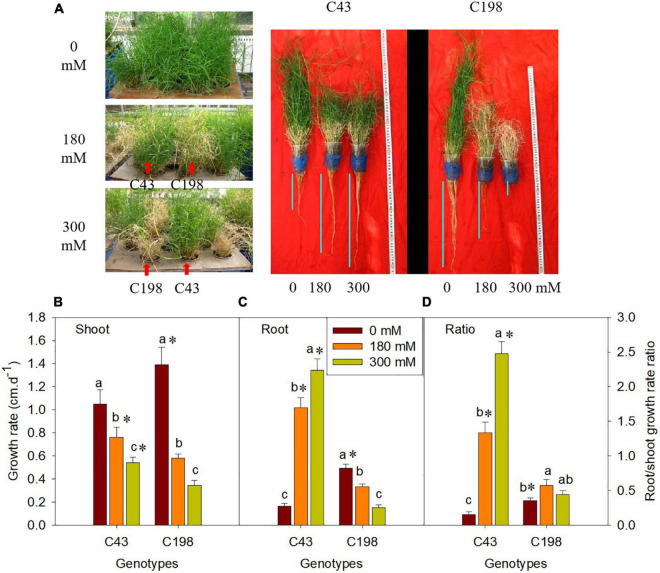
Effect of salt stress on phenotype of bermudagrass (**A**; C43, tolerant genotype; C198, sensitive genotype), and on the shoot growth rate **(B)**, root growth rate **(C)** and root/shoot ratio **(D)** in two genotypes of bermudagrass. Bars marked by the same letters are not significant at *P* < 0.05 (Tukey’s test) for the comparison of different salt concentrations within a given genotype. Bars marked with star (*) indicate significance at *P* < 0.05 (Tukey’s test) between the two genotypes at a given salt concentration. Data represent the mean ± SD of four independent biological replicates.

**FIGURE 2:**
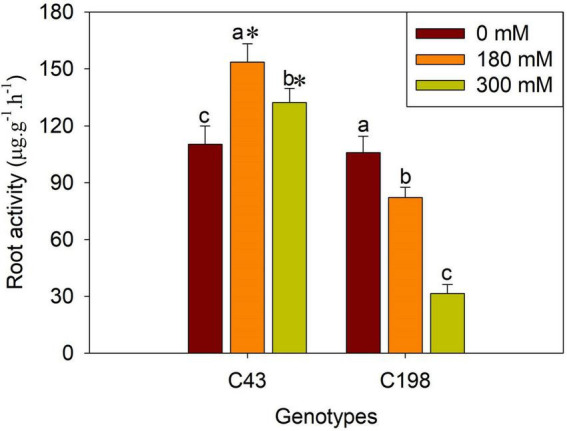
Effects of salt stress on root activity in the two bermudagrass genotypes (C43, tolerant; C198, sensitive). Bars marked by the same letters are not significant at *P* < 0.05 (Tukey’s test) for the comparison of different salt concentrations within a given genotype. Bars marked with star (*) indicate significance at *P* < 0.05 (Tukey’s test) between the two genotypes at a given salt concentration. Data represent the mean ± SD of four independent biological replicates.

Salt stress caused severe damage to shoot growth in both bermudagrass genotypes, but the effect was more pronounced in the ‘C43’ genotype, as evidenced by severe leaf damage and reduced shoot growth (Figure 1). Both genotypes showed a higher shoot growth rate than root growth under normal conditions, and the salt-sensitive genotype had significantly higher shoot and root growth rates than the salt-tolerant (Figure 1). Interestingly, root growth was promoted in the salt-tolerant genotype ‘C43,’ but inhibited in the salt-sensitive ‘C198’ genotype (Figure 1C). Although both genotypes exhibited a high root-to-shoot ratio when exposed to salt stress, the tolerant genotype had a significantly higher root/shoot ratio and root activity than the salt-sensitive genotype up on salt stress (Figures 1D, 2).

### Physiological responses of bermudagrass to salinity stress

**FIGURE 3:**
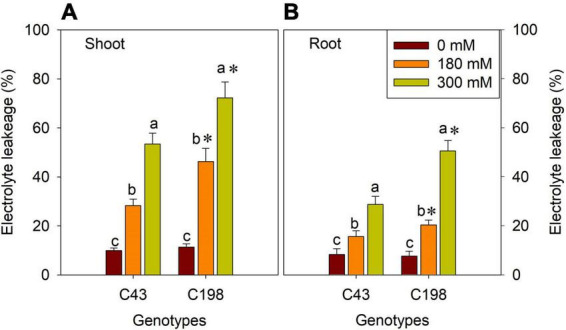
Effects of salt stress on shoot **(A)** and root **(B)** membrane stability in the two bermudagrass genotypes (C43, tolerant; C198, sensitive). Bars marked by the same letters are not significant at *P* < 0.05 (Tukey’s test) for the comparison of different salt concentrations within a given genotype. Bars marked with star (*) indicate significance at *P* < 0.05 (Tukey’s test) between two genotypes at a given salt concentration. Data represent the mean ± SD of four independent biological replicates.

We measured electrolyte leakage in the leaves and roots of the two bermudagrass genotypes to investigate the degree of salt stress-induced membrane damage (Figure 3). The two genotypes did not show a significant difference under control conditions. However, salt stress exposure significantly increased ion leakage in the leaves and roots of both genotypes, more pronounced in the salt-sensitive genotype (Figure 3). Notably, salt stress-induced electrolyte leakage was lower in the roots than in the leaves in both genotypes (Figure 3).

**FIGURE 4:**
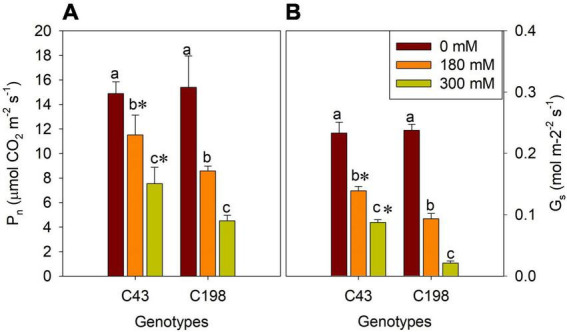
Effects of salt stress on leaf net photosynthetic rate (*P*_n_, **A**) and stomatal conductance (*G*_s_, **B**) in the two genotypes of bermudagrass (C43, tolerant; C198, sensitive). Bars marked by the same letters are not significant at *P* < 0.05 (Tukey’s test) for the comparison of different salt concentrations within a given genotype. Bars marked with star (*) indicate significance at *P* < 0.05 (Tukey’s test) between the two genotypes at a given salt concentration. Data represent the mean ± SD of four independent biological replicates.

Furthermore, to get insights into the effect of salt stress on the photosynthetic process of bermudagrass, we measured net *P*_n_ and *G*_s_ in the leaves of both genotypes after salt stress. There were no significant differences in these parameters between the two bermudagrass under normal conditions. But, salt stress significantly reduced *P*_n_ (Figure 4A) and *G*_s_ (Figure 4B) in both genotypes compared to the control. The decrease in these photosynthetic parameters was aggravated with increasing salt concentration. Interestingly, the salt-tolerant genotype ‘C43’ showed significantly higher *P*_n_ and *G*_s_ than the salt-sensitive bermudagrass, indicating its tolerance to salt stress (Figures 4A,B).

### Hormonal responses to salinity stress

**FIGURE 5:**
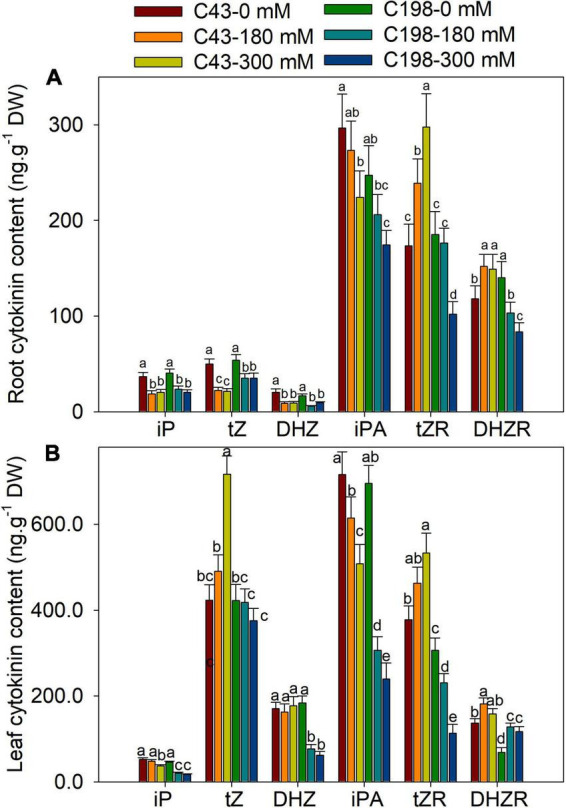
Effects of salt stress on root **(A)** and leaf **(B)** cytokinin content in the two genotypes of bermudagrass (C43, tolerant; C198, sensitive). Bars marked by the same letters are not significant at *P* < 0.05 (Tukey’s test) for the comparison of different salt concentrations in two genotypes. Data represent the mean ± SD of four independent biological replicates.

Given the role of phytohormones in salt tolerance, an HPLC-MS/MS approach was used to quantify the accumulations of various phytohormones in both genotypes after salt exposure. The representative chromatogram of hormone standards and tissue extracts showed that ABA, auxin (IAA, IBA), cytokinins (tZ, tZR, DHZ, DHZR, iP, iPA) and GA_3_ had clear separations (Supplementary Figure 1). Under the control condition, a significant difference was observed for DHZR between the roots of two genotypes (Figure 5A). Salt stress decreased the iP, tZ and DHZ content in the roots of both genotypes compared to the control, and no significant difference was observed between ‘C43’ and ‘C198’ under both salt stress levels. Despite salt stress decreased the content of root iPA in both genotypes, ‘C43’ had significantly higher root iPA content than ‘C198’ at 180 and 300 mM salt treatments. Interestingly, the root tZR content was significantly increased in the salt-treated ‘C43,’ but decreased in ‘C198’ compared to the control (Figure 5A).

Both genotype’s leaf iP and iPA content showed decreasing trends with increasing NaCl concentrations (Figure 5B). Relative to the control, the leaf iP content of ‘C43’ decreased to 91% and 71% and that of ‘C198’ to 45% and 37% under 180 and 300 mM NaCl, respectively. In ‘C43’ bermudagrass, 180 and 300 mM NaCl decreased the leaf iPA content to 85% and 71%, but to 44% and 34% l in ‘C198,’ respectively, relative to the control (Figure 5B). Under control conditions, the leaf tZR and DHZR content were higher in ‘C43’ than ‘C198.’ Salt stress caused a significant decline in leaf DHZ content in ‘C198’ but had no effect in ‘C43.’ Compared to the control, salt stress significantly increased leaf tZR content in ‘C43’ by 1.3- and 1.4-fold under 180 and 300 mM NaCl levels, respectively. In contrast, salt stress significantly decreased leaf tZR content in ‘C198’ to 75% and 37% relative to the control (Figure 5B). Salt stress significantly increased leaf DHZR content in both genotypes, with a higher level in ‘C43’ than ‘C198’ bermudagrass.

**FIGURE 6:**
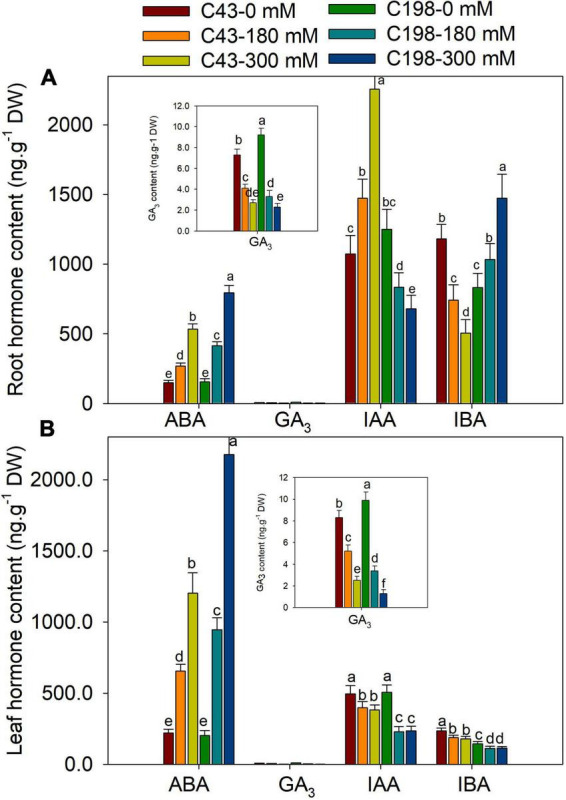
Effects of salt stress on root **(A)** and leaf **(B)** cytokinin content in the two genotypes of bermudagrass (C43, tolerant; C198, sensitive). Bars marked by the same letters are not significant at *P* < 0.05 (Tukey’s test) for the comparison of different salt concentrations in two genotypes. Data represent the mean ± SD of four independent biological replicates.

Salt stress increased the accumulation of ABA in the roots of both genotypes when compared to the control, which was increased by 80% and 267% in ‘C43’ and by 166% and 412% in ‘C198’ after exposure to 180 mM and 300 mM NaCl levels, respectively (Figure 6A). Under the control condition, the root GA_3_ content was higher in ‘C198’ than in ‘C43.’ The root GA_3_ content significantly decreased with salt treatments in both genotypes (Figure 6A). Salt stress caused a significant reduction in leaf IAA content in ‘C198,’ whereas the leaf IAA content significantly increased in ‘C43’ after salt stress. Relative to the control, the root IAA content was increased by 1.4- and 2.1-fold in ‘C43,’ but decreased to 67% and 54% in the salt-sensitive bermudagrass under 180 and 300 mM NaCl levels, respectively (Figure 6A). The root IBA content was higher in ‘C43’ than ‘C198’ under control conditions and showed an opposite trend in both genotypes under salt stress compared to the IAA content. Increasing the salt stress level decreased root IBA content in ‘C43’ and significantly increased in ‘C198’ compared to the control plants (Figure 6A).

Salt stress also induced a remarkable accumulation of ABA in the leaves of both genotypes compared to the control. Exposure of plants to 180 mM and 300 mM NaCl increased ABA content by 2.9- and 5.4-fold in ‘C43’ and 4.6- and 10.7-fold in ‘C198’ relative to the control, respectively (Figure 6B). The salt-sensitive bermudagrass exhibited higher leaf GA_3_ content than the salt-tolerant under control conditions. The leaf GA_3_ content significantly decreased with an increasing salt concentration in both genotypes (Figure 6B). Salt stress caused a significant reduction in leaf IAA content in ‘C198’ but significantly increased in ‘C43’ after salt stress exposure. We observed 1.4- and 2.1-fold increase in leaf IAA content in ‘C43’ under 180 and 300 mM NaCl, respectively. In comparison, leaf IAA content was significantly decreased to 67% and 54% in ‘C198’ after 180 and 300 mM NaCl, respectively (Figure 6B).

Furthermore, correlation analysis was performed to understand the relationship between different traits. The result showed that leaf and root ABA contents were negatively correlated with the shoot growth rate under salt stress. The shoot growth rate was positively correlated with the leaf IAA, GA_3_ and iPA, and root iP and iPA content. The root growth rate was positively correlated with the leaf iP, iPA, tZ and tZR, and root IAA, tZR and DHZR content under salt stress (Table 2).

**TABLE 2:** Linear correlation coefficients between growth parameters.

Leaf hormones		ABA	IAA	IBA	GA_3_	iP	iPA	tZ	tZR	DHZ	DHZR
	**Shoot growth rate**	−0.92*	0.77*	0.17	0.97*	0.035	0.76*	−0.04	0.14	0.56	−0.27
	**Root growth rate**	0.48	0.06	0.10	−0.16	0.74*	0.85*	0.84*	0.61*	0.31	0.19
	**Root/shoot growth ratio**	0.56	−0.07	−0.05	−0.28	0.90*	0.66*	0.90*	0.88*	0.12	0.42
**Root hormones**											
	**Shoot growth rate**	−0.95*	0.04	0.46	0.64	0.82*	0.62*	0.39	0.12	0.25	0.29
	**Root growth rate**	0.02	0.87*	−0.91*	−0.38	−0.06	0.03	−0.39	0.87*	−0.15	0.74*
	**Root/shoot growth ratio**	0.42	0.88*	−0.61	−0.59	−0.47	−0.06	−0.79*	0.55	−0.44	0.27

*Indicate significant correlation at the 0.05 level.

The bold values are also indicating significant correlation at the 0.05 level.

### Hormones related gene expressions in bermudagrass

**FIGURE 7:**
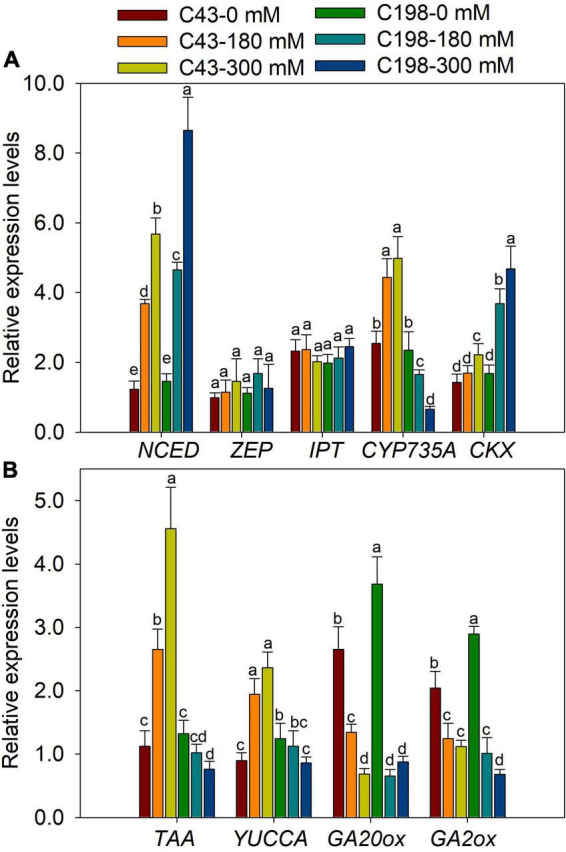
Gene expression levels in roots of the two bermudagrass genotypes differing in salt tolerance (C43: tolerant; C198: sensitive). **(A)** genes related to ABA and cytokinin biosynthesis; **(B)** genes relate to auxin and GA biosynthesis. Bars marked by the same letters are not significant at *P* < 0.05 (Tukey’s test) for the comparison of different treatments. Data represent the mean ± SD of four independent biological replicates.

To further examine whether salt regulation of root and shoot growth was associated with hormonal responses at the transcription level, the expression of genes involved in the biosynthesis of ABA, GA, cytokinins, and IAA were measured using quantitative RT-PCR. We investigated the transcription levels of two ABA biosynthesis genes, Zeaxanthin epoxidase (*ZEP*) and 9-*cis*-epoxycarotenoid dioxygenase (*NCED*). Salt stress did not affect the expression of *ZEP* in both genotypes. However, the expression of *NCED* was markedly increased in the roots of the two bermudagrass genotypes, more profoundly in ‘C198’ than in ‘C43’ (Figure 7A). We also analyzed the expression levels of the isopentenyl transferase (*IPT*) and *CYP735A* genes, encoding the key enzymes involved in the biosynthesis of tZR. Salt stress did not affect the expression level of *IPT* in the roots of both genotypes. Interestingly, *CYP735A* exhibited contrasting expression between the two bermudagrass in response to salt stress, increased in ‘C43’ and decreased in ‘C198’ (Figure 7A). We examined the changes in the expression of genes involved in the degradation of cytokinins (cytokinin oxidase/dehydrogenases, *CKX*). Salt stress caused a remarkable increase in the expression level of *CKX* in ‘C198,’ particularly under severe salt stress (300 mM). In comparison, only a slight increase in the expression level of CKX was observed in ‘C43’ under severe salt stress (300 mM) (Figure 7A).

**FIGURE 8:**
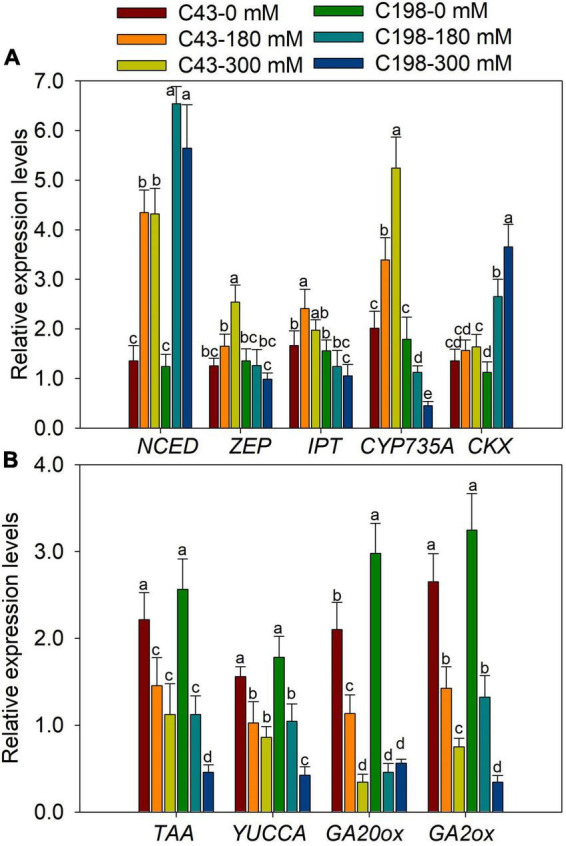
Gene expression levels in leaves of the two bermudagrass genotypes differing in salt tolerance (C43: tolerant; C198: sensitive). **(A)** genes related to ABA and cytokinin biosynthesis; **(B)** genes relate to auxin and GA biosynthesis. Bars marked by the same letters are not significant at *P* < 0.05 (Tukey’s test) for the comparison of different treatments. Data represent the mean ± SD of four independent biological replicates.

The genes encoding the key enzymes for IAA biosynthesis, *TAA* and *YUCCA*, were up-regulated in ‘C43’ and down-regulated in ‘C198’ following salt stress (Figure 7B). The expression level of GA-synthesis genes (*GA20ox*) and GA-degradation genes (*GA2ox*) were significantly down-regulated under salt stress in the roots of the two genotypes (Figure 7B). Salt stress induced the expression of *NCED* in the leaves of the two bermudagrass genotypes, while the *ZEP* was markedly increased in ‘C43’ but decreased in ‘C198’ under salt stress (Figure 8A). Contrastingly, the expression levels of *IPT* and *CYP735A* were increased in the leaves of ‘C43’ but decreased in ‘C198’ after salt stress (Figure 8A). Salt stress increased the expression level of *CKX* only in the leaves of ‘C198’ (Figure 8A). *TAA* and *YUCCA* expression levels were significantly decreased in leaves of both genotypes under salt stress conditions, with a pronounced decrease in ‘C198’ than in ‘C43’ compared to the control (Figure 8B). Similarly, the expression levels of *GA20ox* and *GA2ox* involved in GAs metabolism were significantly decreased with an increasing salt concentration in the leaves of both genotypes (Figure 8B).

## Discussion

The adverse impact of salt stress on plant growth and development and related tolerance mechanisms have been widely reported in various plant species (Zelm et al., 2020; Zhao et al., 2021). However, how plants reprogram different phytohormones in response to salt stress and mechanisms associated with phytohormones-mediated root growth and salt tolerance are poorly understood, especially in bermudagrass. This study investigated the morphological, physiological and hormonal responses of two contrasting bermudagrass genotypes; salt-tolerant ‘C43’ and susceptible ‘C43’ (Hu et al., 2012, 2015).

The deleterious effect of salt stress is intensely associated with osmotic stress and ion toxicity due to the accumulation of toxic ions in plant cells (Munns and Tester, 2008). Increasing evidences revealed that salt stress reduces membrane integrity, water content, enzyme activity, and photosynthesis (Shahid et al., 2020; Fang et al., 2021). When exposed to moderate and high salt stress levels, the two bermudagrass genotypes showed a clear phenotypic difference. Under salt stress, the salt-tolerant bermudagrass ‘C43’ showed better green leaves, membrane stability, and photosynthetic activity than the salt-sensitive ‘C198.’

Plant roots are a primary organ for nutrient and water uptake from the soil. Thus, the root system could play a key role in plant salt tolerance (Zou et al., 2021). The literature revealed that salt stress harms the growth and development of plant roots. Meanwhile, studies also indicated that salt stress could promote root growth in salt-tolerant grasses/halophytes (Meng et al., 2018; Rahman et al., 2021). In the present study, the two genotypes showed a contrasting root growth under salt stress. The root growth of the salt-tolerant bermudagrass ‘C43’ was promoted by salt stress, resulting in the higher root/shoot ratio and root activity. Conversely, the root growth of the salt-sensitive bermudagrass was significantly inhibited by salt stress, leading to a lower root/shoot ratio and diminished root activity.

It is well established that an improved root growth under salt stress could retain more toxic ions and prevent translocation to the shoot, providing a crucial mechanism of salt stress tolerance (Cassaniti et al., 2009). Additionally, better root growth could help plants uptake more water and nutrients from the soil under salt-induced osmotic stress conditions (Zou et al., 2021). Here, the salt-tolerant genotype appeared to allocate more energy to root growth, as evidenced by promoted root growth in response to salt stress, which could be an adaptive response to recover the functional equilibrium under new environmental conditions (Li and Li, 2017). Overall, the better green leaves, membrane stability, and higher photosynthetic activity in ‘C43’ could result from improved root growth that retains more toxic ions and/or maintains the water and nutrient supply to the shoots.

### Cytokinin is involved in bermudagrass root growth and salt tolerance

**FIGURE 9:**
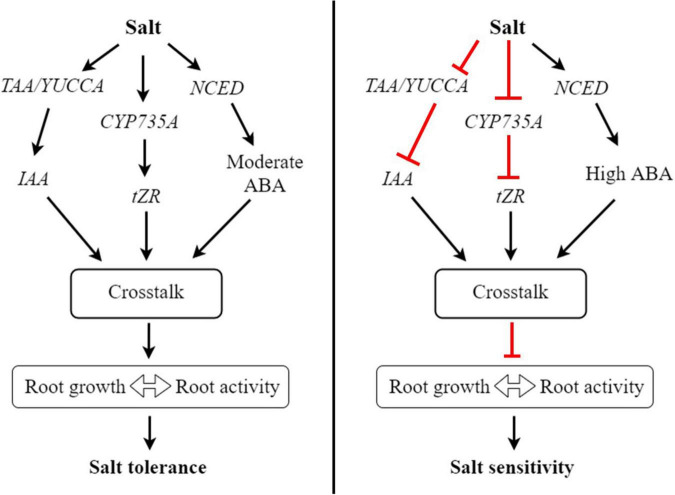
A proposed working model of how hormonal reprogramming and crosstalk are involved in root growth and salt tolerance in bermudagrass. In roots, salt stress induces the expression of *TAA/YUCC, CYP735A*, and *NCED* genes involved in auxin, cytokinin, and ABA biosynthesis, respectively. The activation of these genes increases the accumulation of respective hormones in roots. Subsequently, these hormones could work together through hormonal crosstalk and increase root growth and root activity, ultimately improving salt tolerance in bermudagrass. In contrast, salt stress inhibits the expression of *TAA/YUCC* and *CYP735A* genes, leading to reduced auxin and cytokinin levels in roots, respectively. On the other hand, salt stress strongly induces the expression of *NCED*, leading to the accumulation of a high level of ABA in roots. The reduced levels of auxin and cytokinin and high ABA level undergo hormonal crosstalk in roots and inhibit root growth, thus diminishing root activity and increasing salt sensitivity in bermudagrass.

Cytokinin is the most crucial hormone that plays a crucial role in the plant cell cycle and various developmental processes, including cell division, cell elongation, transportation of nutrients, root growth, and abiotic stress response (Wu et al., 2021). In this study, the three derivatives of CK, tZ, tZR and DHZR were notably increased in the salt-tolerant genotype but decreased in the salt-sensitive genotype, suggesting that CK may play roles in bermudagrass root growth and salt stress tolerance. Similar results have been reported in *Suaeda salsa* under salinity stress (Guo et al., 2020). *Adenosine phosphate-isopentenyl transferase (IPT)* is a key enzyme that catalyzes the *de novo* synthesis of CKs and the biosynthesis of the major derivatives of CKs, iP- and tZ is catalyzed by *IPT*. Overexpression of *IPT* has enhanced the biosynthesis of CKs (Galichet et al., 2008). In our study, the expression of *IPT* was notably upregulated in the leaves of ‘C43’ genotype, which could be attributed to the higher accumulation of tZ in ‘C43’ leaves. But, *IPT* was decreased in ‘C198,’ resulting in a decrease in tZ content. *CK oxidase/dehydrogenases (CKX)*, an enzyme that catalyzes the degradation of CK, was upregulated by salt stress in both genotypes, but ‘C198’ had significantly higher expression in both leaves and roots, resulting in lower CK contents in both tissues. The upregulation of *IPT* in the salt-tolerant bermudagrass was responsible for the accumulation of CK, especially tZ, and may be involved in the salt tolerance of bermudagrass (Figure 9).

*CYP735A* is one of the critical enzymes involved in the CK biosynthesis pathway and is required for shoot growth in Arabidopsis (Kiba et al., 2013). Mutation of *CYP735A* significantly reduced the contents of tZ and tZR, leading to a pronounced growth defect in Jatropha plants (Cai et al., 2018). In our study, the concentration of tZR was significantly higher in the roots of ‘C43’ genotype, suggesting that the accumulation of this CK derivative could play a significant role in bermudagrass salt tolerance by promoting root growth. Interestingly, the expression of *CYP735A* was remarkably increased in the roots of ‘C43,’ but decreased in ‘C43’ after salt stress. Collectively, these results demonstrate that the upregulation of crucial CK biosynthesis genes could play an invaluable role in salt tolerance by increasing the accumulation of CK derivatives, thereby promoting root growth and salt tolerance in bermudagrass (Figure 9).

### The role of auxin in root growth and salt tolerance in bermudagrass

Auxin is a critical plant hormone involved in growth, development, and stress response (Zhao, 2018; Blakeslee et al., 2019; Cao et al., 2019). Auxin can be synthesized through the *tryptophan aminotransferase (TAA)* and *YUCCA (YUC)* enzymes-mediated (TAA)/YUCCA (YUC) pathway. In Arabidopsis, the inactivation of several YUC genes dramatically decreased endogenous IAA (Chen et al., 2014). Additionally, the endogenous level of IAA in *yuc1 yuc2 yuc6* triple mutants was decreased, leading to increased drought sensitivity in Arabidopsis (Shi et al., 2014). In this study, the concentration of IAA in the roots of the salt-tolerant bermudagrass ‘C43’ was significantly higher than that of ‘C43’ after salt exposure, suggesting that IAA could play a role in root growth. Similarly, salt stress-induced accumulation of IAA has been reported in the roots of a halophyte species, *Prosopis strombulifera* (Llanes et al., 2014).

On the other hand, salt stress reduced the expression of *TAA* and *YUCCA* genes in the leaves of both genotypes. Interestingly, the expression levels of these genes were dramatically increased in the roots of ‘C43,’ but conversely reduced in ‘C198,’ which was consistent with the higher accumulation of IAA in ‘C43.’ It is well established that the higher expression of auxin biosynthesis genes results in higher IAA production in root tips (Brumos et al., 2018). Accumulation of IAA promoted root growth in tea plants exposed to aluminum stress (Gao et al., 2022). Thus, we can speculate that root growth and salt tolerance in bermudagrass could be related to auxin accumulation in the root due to the increased expression of *TAA* and *YUCC* genes by salt stress, as shown in the model (Figure 9).

### Abscisic acid function in root growth and salt tolerance in bermudagrass

It is well documented that salt-induced osmotic stress could increase the concentration of ABA in both roots and shoots of salt-treated plants, providing a mechanism of salt stress tolerance (Ryu and Cho, 2015; Yu Z. et al., 2020; Huang et al., 2021). Here, salt stress increased the ABA content in the leaves and roots of both genotypes, more prominently in the salt-sensitive genotype. *Zeaxanthin epoxidase (ZEP)* and *9-cis-epoxycarotenoid dioxygenase (NCED)* are critical enzymes in the biosynthesis of ABA. In this study, salt stress did not affect the expression of ZEP in the roots of both genotypes.

Additionally, the expression of *NCED* was induced by salt stress in bermudagrass. The expression was significantly higher in the roots and leaves of the C198 genotype, consistent with the elevated levels of ABA in both tissues of the salt-sensitive genotype. The accumulation of ABA is primarily associated with the upregulation of *NCED* genes (Leng et al., 2013). Thus, the accumulation of ABA in bermudagrass is related to the pronounced expression of *NCED* by salt stress (Figure 9). Because ABA is often recognized as a negative regulator of plant growth (Brookbank et al., 2021), the reduction of shoot growth in the two bermudagrass could have resulted from the increased accumulation of endogenous ABA in the salt-treated leaves.

Interestingly, the increased ABA content was positively correlated with the reduced shoot growth (Table 2). More importantly, ABA regulates root growth and lateral root branching in plants (Sah et al., 2016; Wang et al., 2017). ABA was reported to promote root growth at low concentrations, but it could inhibit root formation at high concentrations (Rowe et al., 2016; Li et al., 2017). In the present study, a higher concentration of ABA was observed in the salt-sensitive genotype with retarded root growth and poor root activity. The increased levels of ABA in the roots and leaves of ‘C198’ could be one reason for the dramatic inhibition of shoot and root growth, increased salt sensitivity.

### The role of gibberellin in bermudagrass root growth and salt tolerance

Gibberellin is crucial for plant growth and development, and its deficiency is associated with plant dwarfism (El-Sharkawy et al., 2012; Lee et al., 2014). Stress conditions could inhibit GA biosynthesis and/or increase its degradation, disturbing normal plant metabolism and growth (Llanes et al., 2016). Here, the two bermudagrass genotypes showed a reduced level of GA_3_ in response to salt stress, leading to shoot growth reduction. *GA20-oxidase (GA20-ox)* and *GA2-oxidase (GA2ox)* are key enzymes involved in the biosynthesis and degradation of GAs, respectively. Overexpression of *OsGA2ox5* improved rice tolerance to salt stress (Shan et al., 2014).

*GA20ox2* was involved in root elongation and root branching by regulating IAA synthesis and transport in Arabidopsis (Lv et al., 2018). Thus, the decrease in GA_3_ in bermudagrass could be related to the downregulation of *GA2ox* and *GA20ox* by salt stress. Under normal conditions, the GA_3_ content was higher in the salt-sensitive genotype, but relatively ‘C43’ had significantly higher GA_3_ content under salt stress. At optimal concentration, GA_3_ is beneficial for the physiology and metabolism of plants under abiotic stresses (Iqbal et al., 2011). These results suggest that the salt-tolerant genotype could produce optimum GA_3_ to maintain normal functioning, thus improving root growth and salt tolerance.

### Hormonal crosstalk is essential for root growth, shoot source activity and salt tolerance in bermudagrass

When exposed to a stressful condition, plants reprogram the biosynthesis and transport of hormones in a way to cope with the stress. Hormonal crosstalk is a crucial process for the understanding of the role of phytohormones, because hormones may not act independently to regulate plant growth and development (Pacifici et al., 2015). To provide a stress defense mechanism and regulate root development, hormonal crosstalk could occur at multiple levels (Murphy, 2015; Aerts et al., 2020). During lateral root formation, cytokinin has been reported to play antagonistic role with auxin in regulating shoot branching (Shimizu-Sato et al., 2009), and lateral root growth (Durbak et al., 2012).

**FIGURE 10:**
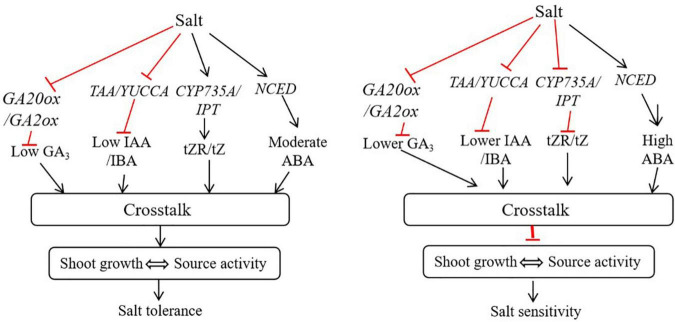
A proposed working model of how hormonal reprogramming and crosstalk are involved in shoot growth and salt tolerance in bermudagrass. In shoots, salt stress induces the expression of CYP735A/IPT and *NCED* genes involved in tZR, tZ and ABA biosynthesis. The activation of these genes increases the accumulation of respective hormones in shoots. Subsequently, these hormones could work together through hormonal crosstalk and maintain shoot growth and source activity, ultimately improving salt tolerance in bermudagrass. In contrast, salt stress inhibits the expression of *TAA/YUCC*, *CYP735A/IPT*, and *GA20ox/GA2ox* genes, leading to reduced auxin, cytokinin and gibberellin levels in shoot, respectively. On the other hand, salt stress strongly induces the expression of *NCED*, leading to the accumulation of a high level of ABA in shoot. The reduced levels of auxin, gibberellin and cytokinin and high ABA level undergo hormonal crosstalk in shoot and inhibit shoot growth, thus diminishing source activity and increasing salt sensitivity in bermudagrass.

Studies have shown that the crosstalk and regulation of hormones by each other occurs at the transcription level through regulating biosynthesis and degradation genes. For instance, auxin could inhibit *IPT* expression and conversely increase the expression of *CKX* to inhibit CK biosynthesis (Nordstrom et al., 2004). Despite they work antagonistically at low and moderate concentrations however, they auxin and CK can work synergistically at higher concentration (Kurepa et al., 2019). In our study, the contents of auxin and cytokinin were higher in the salt tolerant genotype, suggesting that these hormones may work together to positively regulate root growth and shoot source activity in bermudagrass under salt stress (Figures 9, 10). Additionally, the expression of *IPT* was higher and CKX was lower in the ‘C43,’ suggesting that auxin may not antagonistic to CK in bermudagrass.

The negative effect of ABA in seed germination was improved by GAs through antagonistic crosstalk (Verma et al., 2016). In this study, the adverse effect of accumulated ABA in salt tolerant genotype bermudagrass could be masked by accumulation of GAs through hormonal crosstalk, leading to improved growth and salt tolerance (Figures 9, 10). The increase in root growth rate during stress can be regulated by ABA, osmotic stress-induced ABA accumulation could induce auxin transport, thereby improve root elongation under osmotic stress (Xu et al., 2013). Here, the moderately increased level of ABA as a result of salt-induced osmotic stress could be involved in auxin transport to regulate root growth and shoot source activity in bermudagrass. CKs can interact with other hormones, including auxin (IAA) and ABA, to regulate the growth and development of roots (Nguyen et al., 2018). Treatment of plants with exogenous IAA and GA3 could improve tolerance to salt stress by regulating various physiological and biochemical traits (Khalid and Aftab, 2020).

The contents of IAA, GA3 and IBA were decreased in the leaves of the two bermudagrass in response to salt stress. Interestingly, IAA, GA3 and IBA contents were significantly higher in ‘C43.’ Moreover, we found a significant positive correlation between the contents of these hormones and shoot growth under control and salt stress conditions (Table 2). Thus, the decrease in shoot growth in both genotypes could be attributed to the decrease in these hormones. The negative effect of increased ABA level in the salt-tolerant genotype could be masked by other hormones through hormonal crosstalk, leading to better shoot growth and salt tolerance. Therefore, the crosstalk between different phytohormones could be crucial in bermudagrass root growth and salt tolerance.

Due to the complex nature of plant hormone interactions, integrating physiological and molecular levels to the whole plant response is a key to uncovering the detailed mechanisms of hormonal crosstalk (Ross et al., 2011). It has been suggested that combining experimental and modeling studies is a crucial way to dissect the complexity of hormonal crosstalk in regulating root development (Liu et al., 2014). In maize, a higher level of ABA resulting from salt stress has been shown to disturb auxin transport and distribution and inhibit lateral root growth (Lu et al., 2019). A higher salt level could lead to a higher ABA accumulation, which negatively regulates auxin distribution and root development, while moderate salt stress has the opposite effect (Yu Z. et al., 2020). Therefore, in bermudagrass, the decrease in root growth could be associated with a significantly higher level of ABA caused by salt stress, which further inhibits auxin biosynthesis and distribution.

## Conclusion

This study demonstrates the role of hormonal reprogramming and crosstalk in regulating root growth and salt tolerance in bermudagrass. In addition, to the morphological and physiological differences, the two contrasting genotypes showed an apparent hormonal regulation, including biosynthesis and degradation in response to salt stress. With improved root growth and root activity by salt stress, the salt-tolerant genotype ‘C43’ had a higher accumulation of IAA, CK, and GA_3_ in the roots, which are crucial for plant growth and development. The expressions of hormones related genes involved in the biosynthesis of these hormones were induced by salt stress in the salt-tolerant genotype. The antagonistic interaction of two hormones might be affected by the third hormone to synergistically improve root growth and salt tolerance in bermudagrass. Moreover, the crosstalk of hormones and the outcomes depend on the stress condition, the plant species, and the hormones concentration. Thus, further study is necessary to elucidate how hormonal crosstalk regulates root growth and salt tolerance in bermudagrass at the molecular level.

## Data availability statement

The raw data supporting the conclusions of this article will be made available by the authors, without undue reservation.

## Author contributions

YY, QX, and L-XH conceived and designed the experiments. YY and MW performed the experiments and wrote the manuscript. YY, MW, and N-FL analyzed the data. HD and Y-BZ helped with manuscript reviewing. QX and L-XH revised the manuscript. All authors read, revised and approved the final manuscript.

## Supplementary material

Supplementary Figure 1 Representative chromatogram of hormone standards and tissue extracts of bermudagrass under salt stress **(A)** cytokinin standards; **(B)** cytoinin in tissues; **(C)** ABA, auxin andGA3 standards; **(D)** ABA, auxin and GA3 in tissues.

## Abbreviations

ABA: abscisic acid
DHZ: dihydrozeatin
GA: gibberellic acid
iP: isopentenyl
tZ: *trans*-zeatin
iPA: isopentenyl adenosine
DHZR: dihydrozeatin riboside
IAA: indole acetic acid
tZR: *trans*-zeatin riboside
*P*_n_: net photosynthetic rate.
