# Supplementary figures and images for: Genotypic-specific hormonal reprogramming and crosstalk are crucial for root growth and salt tolerance in bermudagrass (Cynodon dactylon)
Source: Front Plant Sci. 2022 Aug 4;13:956410. doi: 10.3389/fpls.2022.956410 (PMC9386360; doi:10.3389/fpls.2022.956410)

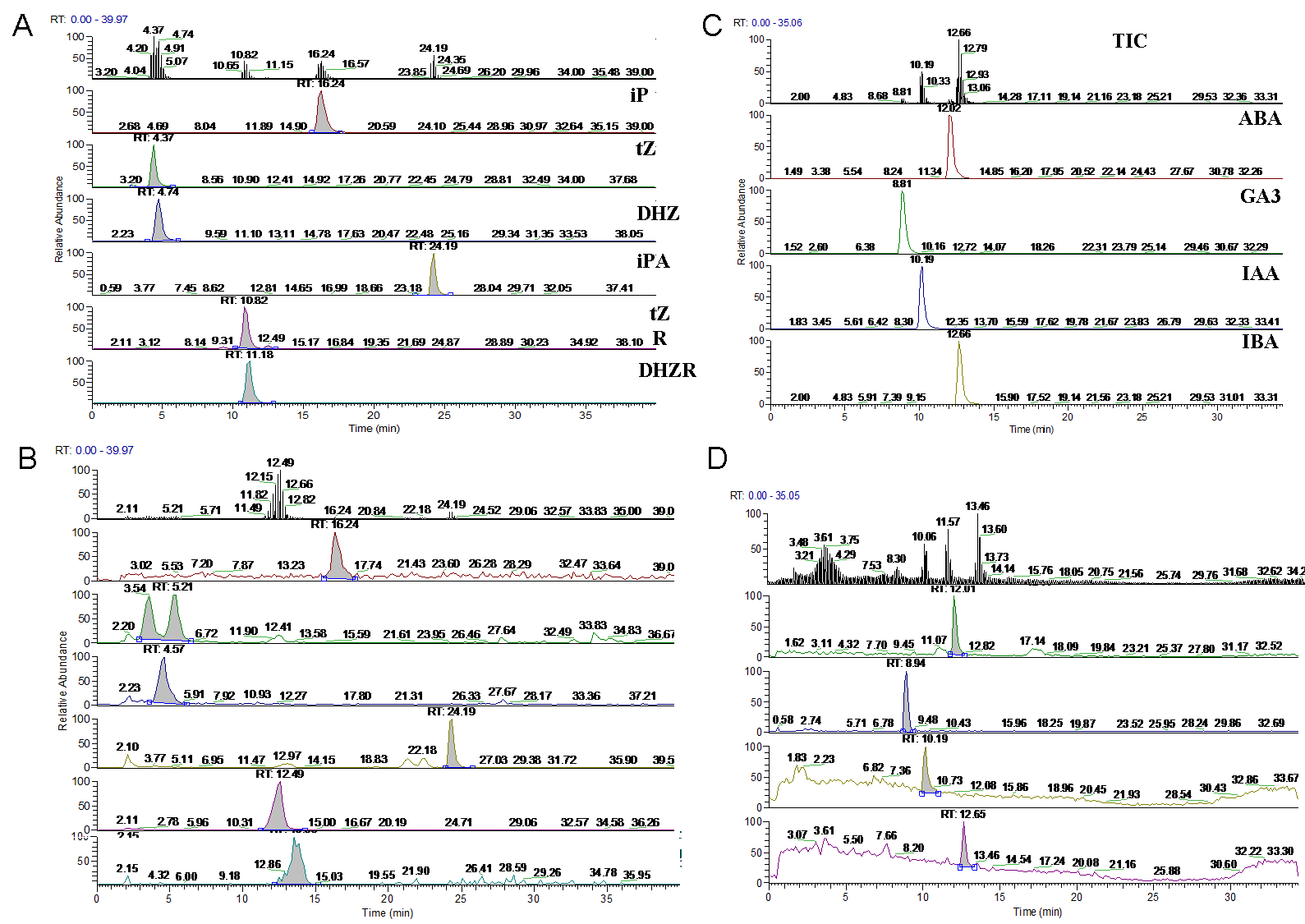

Supplement: Supplementary Figure 1 — Representative chromatogram of hormone standards and tissue extracts of bermudagrass under salt stress (A) cytokinin standards; (B) cytoinin in tissues; (C) ABA, auxin andGA3 standards; (D) ABA, auxin and GA3 in tissues. [file Image_1.TIF]
